# Evaluation of a short formation on the performance of point-of-care renal ultrasound performed by physicians without previous ultrasound skills: prospective observational study

**DOI:** 10.1186/s13089-017-0078-8

**Published:** 2017-11-09

**Authors:** François Javaudin, François Mounier, Philippe Pes, Idriss Arnaudet, Frédéric Vignaud, Eric Frampas, Philippe Le Conte, Winfocus-France study group

**Affiliations:** 1 Service des urgences, Centre Hospitalier Universitaire de Nantes, 44035 Nantes cedex, Nantes, France; 2 Service de radiologie, Centre Hospitalier Universitaire de Nantes, Nantes, France

**Keywords:** Point-of-care ultrasound, Hydronephrosis, Training, Evaluation

## Abstract

**Background:**

Point-of-Care Ultrasound (PoCUS) is recommended by emergency medicine societies for the detection of hydronephrosis. Training of certified Emergency Physicians (EP) without prior ultrasound experience remains debated. We investigate performance of a brief training session for the detection of hydronephrosis with PoCUS performed by EP without previous ultrasound experience.

**Patients and methods:**

This was a prospective observational study of a convenience sample of patients older than 18 years with presumed renal colic, acute pyelonephritis or documented acute renal failure. Exclusion criteria were pregnancy and documented end of life.After inclusion and informed consent, a PoCUS was performed. A radiologist’s renal ultrasound (RRUS) was then conducted, the radiologist being blind to PoCUS result.The objective was to determine the diagnostic performance of PoCUS performed by EP for the detection of hydronephrosis using RRUS as gold standard.

**Results:**

Six EP participated in this study. 55 patients were included, five secondary excluded for lack of RRUS. Age was 47 ± 22 years, sex ratio 1. Hydronephrosis prevalence was 38% (CI 95% [26–52%]). Sensitivity of PoCUS was 100% (CI 95% [82–100%]) while its specificity was 71% (CI 95% [52–86%]) with a NPV of 100% (CI 95% [85–100%]) and a 68% (CI 95% [48–84%]) PPV. Kappa coefficient was 0.65 (CI 95% [0.45–0.85]).

**Discussion:**

We demonstrated that a short training program enables EP without previous ultrasound skills to rule out hydronephrosis with satisfactory performances. The main limitation was the absence of collection of the number of PoCUS by EP. After this didactic course, an experiential phase must be carried out.

## Introduction

A renal ultrasound (RUS) is mandatory in suspected renal colic (RC), acute pyelonephritis (AP) and acute renal failure (ARF) in search of hydronephrosis because management would be altered. A review on point-of-care ultrasound (PoCUS) found that sensitivity ranged from 72 to 97% and specificity from 73 to 83% for the presence of hydronephrosis [3]. Emergency Medicine Societies promote usage of PoCUS in suspected renal colic [1, 4, 10].

The training of certified Emergency Physicians (EP) with no prior ultrasound (US) experience remains debated. In our institution, such EP participates to a 16-h training program over a 2-day period (USLS-BL1 endorsed by WINFOCUS International). The program includes operation of ultrasound device, interpretation of normal and pathological images to assess hydronephrosis, free peritoneal and pericardial fluid, proximal deep venous thrombosis, pulmonary and first-grade cardiac ultrasound. Approximately half of the time is spent performing imaging under supervision. The aim of this prospective observational survey was to assess the accuracy of renal PoCUS after this course compared with radiologist’s RUS (RRUS) as a gold standard.

### Patients and methods

This was a prospective study of a convenience sample of patients with presumed RC, AP or documented ARF. It was undertaken in the ED of a tertiary teaching hospital with an annual census of 75,000 from August 2014 to March 2015. This study was approved by the Ethics Committee of Nantes University Hospital (reference RC15_0443).

The inclusion criteria for patients were a suspected RC, AP or documented ARF in patients older than 18 years of age. Exclusion criteria were pregnancy, RRUS nearly completed, documented end of life precluding further investigation. Patients for whom RRUS was not performed were secondarily excluded.

Participating EP were recruited in our ED. Inclusion criteria were the absence of previous POCUS exposure before participation to our study, in particular, no POCUS course during their medical school nor during their EM residency. They committed themselves to not follow another POCUS training until conclusion of the study.

After inclusion, information and consent to participate, a PoCUS was performed. A RRUS was then realized, the radiologist being blind to PoCUS result. Only the RRUS result was used for the management of the patients.

Using a Philips CX50 (Philips, Netherlands) with a 3.5–5 MHz curved array probe, EP obtained images of both kidneys. They completed a reporting form including demographic data, the presence or absence of hydronephrosis for each kidney. It was defined as a dilatation of the collective system. Finally, the difficulty for the PoCUS was assessed.

Formal RRUS was performed by radiologist with usual devices in the radiology department. A report was then filled with the same items.

The objective of this study was to assess the accuracy of renal PoCUS after a brief course compared with radiologist’s RUS (RRUS) as a gold standard. As a part of our policy, computed tomography is not performed in this clinical setting. The main objective was sensitivity and negative predictive value (NPV) of PoCUS. Secondary objectives were concordance explored by Kappa coefficient, specificity, positive predictive value (PPV) and likelihood ratios. The required number of subjects for sensitivity 0.9 with alpha risk 0.05 and beta 0.10 was 38.

Values stored in Microsoft Excel™ were analyzed with Graphpad™. 95% confidence intervals were calculated for sensitivity, specificity, NPV, PPV, likelihood ratios and concordance.

## Results

**Fig. 1:**
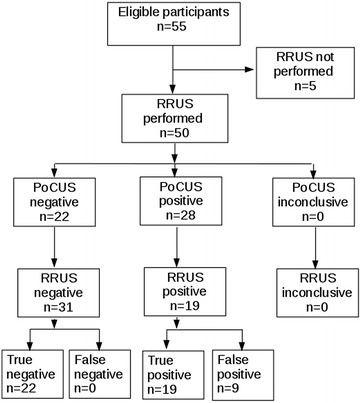
STARD chart of patients included in a prospective study exploring PoCUS accuracy

Six EP participated to this study, four women and two men, mean age 37 ± 7 years old. Mean time since their certification in Emergency Medicine was 7 ± 7 years. 55 patients were included, five secondarily excluded because of lack of RRUS (Fig. 1). Age was 47 ± 22 years, sex ratio 1. There were 31 RC, 9 AP and 10 ARF. Hydronephrosis prevalence was 38% [26–52%]. Sensitivity and NPV were 100% [79–100%] and 100% [81–100%], respectively. Specificity and PPV were 68% [47–83.4%] and 71% [53–81%], respectively. Positive likelihood ratio was 3.4 [2.0–6.0], negative likelihood ratio was 0.0 [0.0–NC] and Kappa coefficient was 0.65 [0.45–0.85] (Table 1). PoCUS difficulty was assessed as difficult (two patients, 4%), medium (13 patients, 26%) or easy (35 patients, 70%).

**Table 1:** Results of PoCUS compared with RRUS for hydronephrosis in 50 patients

	RRUS +	RRUS −
PoCUS +	19 (38%)	9 (18%)
PoCUS −	0 (0%)	22 (44%)
	Sensitivity: 100% [82–100%] NPV: 100% [85–100%]	Specificity: 71% [52–86%] PPV: 68% [48–84%]

*PoCUS* point-of-care ultrasound; *RRUS* radiologist’s renal ultrasound; *NPV* negative predictive value; *PPV* positive predictive value

## Discussion

We found that this training enabled EP without previous US skills to exclude hydronephrosis with good performance. Indeed, all hydronephrosis on RRUS were detected by POCUS performed by EP (*n* = 19) and none (*n* = 0) was detected by RRUS when POCUS was negative. That is why sensitivity and NPV were 100%. However, specificity and PPV were not sufficient. Kappa value indicated a good strength of agreement.

Limitations were the absence of collection of the number of PoCUS by EP and the absence of collection of the delay between PoCUS and RRUS.

The choice of sensitivity as the primary objective was motivated by the fact that hydronephrosis is rare both in ARF [7], AP [2] and is present in only 18% in ED’s acute flank pain patients [9]. Thus, the ability of an EP to rule out hydronephrosis should be more helpful than to confirm its presence. PoCUS might be seen as a screening tool with a high sensitivity and NPV.

Training in ultrasound of certified EP is a challenging problem since many physicians does not use it in every day practice. In USA, a survey performed in Connecticut in 2014 showed that 24% used PoCUS on a daily basis [5]. In Europe, there is a lack of such information but the situation might not be quite different. However, PoCUS is now strongly recommended by Emergency Medicine Societies [1, 4, 10] and is an integrate part of the Emergency medicine curriculum [10]. The American College of Emergency Physicians has formalized the training pathway for EP without previous ultrasound skills [1]. It begins with a didactic course followed by an experiential phase of supervised ultrasounds. A similar PoCUS training pathway is proposed in United Kingdom [10]. Performance of short training period has been investigated in a Spanish study [8] with similar results as ours. The learning curve in the detection of hydronephrosis has been evaluated, the best results were obtained after 30 exams [6].

In conclusion, we demonstrated that a short training program allows EP without previous US skills to rule out hydronephrosis with good performances. The experiential phase of supervised ultrasounds must be carried out.
